# Towards a Synthetic Biology Toolset for Metallocluster Enzymes in Biosynthetic Pathways: What We Know and What We Need

**DOI:** 10.3390/molecules26226930

**Published:** 2021-11-17

**Authors:** Helena Shomar, Gregory Bokinsky

**Affiliations:** 1 INSERM U722, Faculté de Médecine, Université de Paris, Site Xavier Bichat, 75018 Paris, France; 2 Department of Bionanoscience, Kavli Institute of Nanoscience, Delft University of Technology, 2629 HZ Delft, The Netherlands

**Keywords:** metallocluster enzymes, synthetic biology, metabolic engineering, heterologous expression, FeS cluster, electron transfer, enzyme maturation, microbial biosynthesis

## Abstract

Microbes are routinely engineered to synthesize high-value chemicals from renewable materials through synthetic biology and metabolic engineering. Microbial biosynthesis often relies on expression of heterologous biosynthetic pathways, i.e., enzymes transplanted from foreign organisms. Metallocluster enzymes are one of the most ubiquitous family of enzymes involved in natural product biosynthesis and are of great biotechnological importance. However, the functional expression of recombinant metallocluster enzymes in live cells is often challenging and represents a major bottleneck. The activity of metallocluster enzymes requires essential supporting pathways, involved in protein maturation, electron supply, and/or enzyme stability. Proper function of these supporting pathways involves specific protein–protein interactions that remain poorly characterized and are often overlooked by traditional synthetic biology approaches. Consequently, engineering approaches that focus on enzymatic expression and carbon flux alone often overlook the particular needs of metallocluster enzymes. This review highlights the biotechnological relevance of metallocluster enzymes and discusses novel synthetic biology strategies to advance their industrial application, with a particular focus on iron-sulfur cluster enzymes. Strategies to enable functional heterologous expression and enhance recombinant metallocluster enzyme activity in industrial hosts include: (1) optimizing specific maturation pathways; (2) improving catalytic stability; and (3) enhancing electron transfer. In addition, we suggest future directions for developing microbial cell factories that rely on metallocluster enzyme catalysis.

## 1. Introduction

In the two decades since its conception, the field of synthetic biology has moved genetic engineering considerably closer to an actual engineering discipline. The combination of synthetic biology tools with the principles of metabolic engineering have tremendously advanced our ability to engineer microorganisms to produce valuable products [1]. This progress is evident in the gradual shift of production of select commodities and fine chemicals to favor bio-based processes [2,3]. Central to this approach is the use of heterologous hosts as biosynthetic platforms, such as *Escherichia coli* [4], Streptomycetes [5] or yeast [6]. A classic synthetic biology approach to improve microbial biosynthesis commonly involves cloning codon-optimized heterologous genes into the platform host, followed by engineering-controlled expression/balancing of pathway enzymes, and optimizing supplies of pathway precursors and cofactors [7]. However, while synthetic biology has had some success in making *genes* universally compatible with heterologous hosts, far fewer tools have been developed that can adapt *enzymes* to foreign hosts. If enzymes lose catalytic activity when expressed in heterologous hosts, the gene-focused tools of synthetic biology cannot engineer microbial production.

Metallocluster enzymes, one of the most ubiquitous and versatile families of metalloproteins, employ metal-containing cofactors to catalyze some of the most remarkable reactions in nature [8,9]. These enzymes are of great biotechnological relevance as they are involved in the production of valuable natural products—such as pharmaceuticals or biofuels—or in metabolic pathways that utilize alternative carbon sources. However, their application in heterologous biosynthesis is limited, as metallocluster-containing enzymes often display little-to-no activity when expressed in heterologous organisms, leading to low product yields. Consequently, they are a significant bottleneck in the microbial production of industrially important chemicals. This is notably the case for numerous enzymes that contain iron-sulfur (FeS) clusters, which are required for the biosynthesis of a wide variety of useful chemicals. Unfortunately, these enzymes often display limited activity in foreign hosts, hence precluding the development of many relevant microbial cell factories.

The poor activity of metallocluster-containing enzymes in heterologous organisms is often considered to stem from their dependence on poorly characterized essential supporting pathways for their maturation and catalytic cycle. Therefore, traditional metabolic engineering approaches may be blind to the challenges in reconstituting and optimizing metabolic pathways that rely on these enzymes. In this review, we highlight the biotechnological relevance of metallocluster-containing enzymes and the challenges to their application in microbial biosynthesis. Finally, we detail successful engineering strategies that enable the functional expression of metallocluster enzymes to improve recombinant pathway yields for microbial cell factories, with an emphasis on FeS cluster enzymes.

## 2. Biotechnological Relevance of Metallocluster Enzymes: Potential and Challenges

### 2.1. Many Industrially Important Pathways Rely on Metallocluster Enzyme Activity

**Figure 1:**
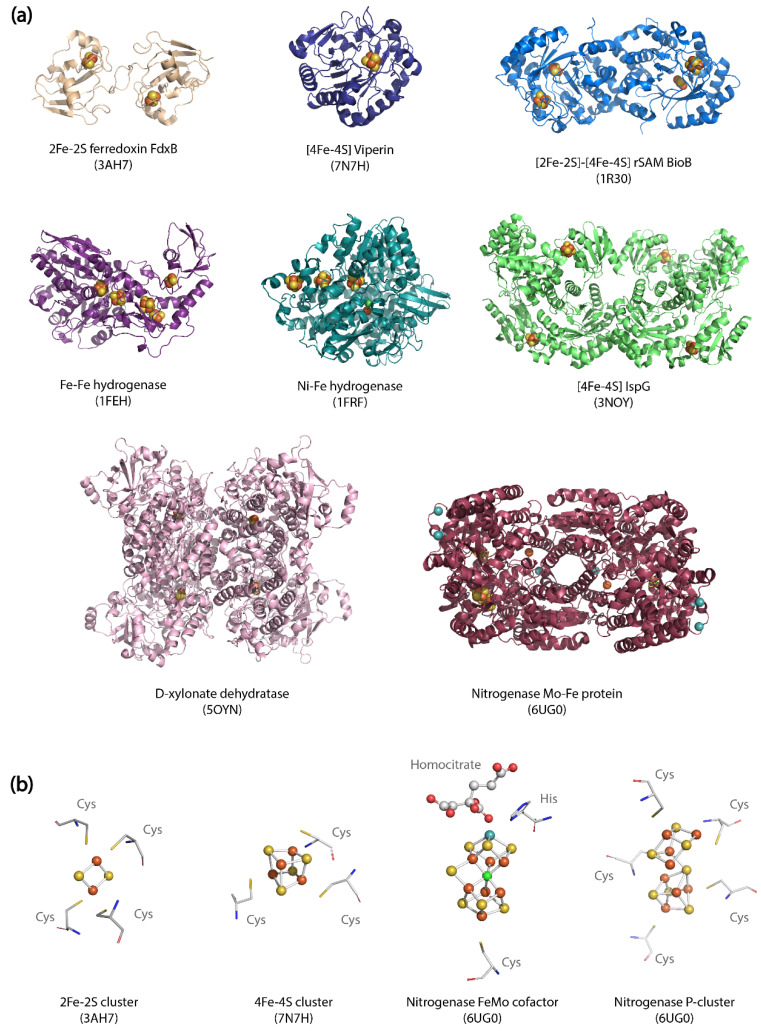
Structural diversity of metallocluster enzymes. (**a**) Crystal structures of diverse metallocluster enzymes bound to their metal cofactors. PDB entries and origin of each protein structures are listed in Appendix A Table A1. (**b**) Structure and environment of different metalloclusters from biotechnologically relevant enzymes.

Enzymes that contain metallocofactors composed of homo- or hetero-nuclear clusters (i.e., [Fe-Fe], [Fe-Ni], [Fe-S] and [Fe-Mo] clusters) [8] are involved in a wide array of metabolic pathways. Metalloclusters participate in reactions either directly by providing catalytic sites, or by acting as electron transport centers. The absence or incomplete maturation of metalloclusters completely eliminates enzymatic activity. The natural ability of metalloclusters to bind a wide range of protein structures also accounts for their biosynthetic versatility (Figure 1). Because metallocluster-containing enzymes facilitate a huge variety of reactions that include oxidative transformations, epimerization, hydroxylation, epoxidation, methyl transfers, alkylation or oxygenation, they hold a tremendous potential for microbial biosynthesis [9,10]. Table 1 highlights metabolic pathways of prominent biotechnological relevance that rely upon reactions catalyzed by metallocluster enzymes.

### 2.2. Challenges Related to Recombinant Pathways That Contain Metallocluster Enzymes

The functional expression of recombinant metallocluster enzymes is often challenging. The abundance of functional metalloenzyme relies on the efficient synthesis and incorporation of metalloclusters into inactive apoenzymes. This maturation process usually involves complex post-translational modifications coordinated by specialized proteins. The proper maturation and incorporation of every metallocluster is essential for catalysis. Furthermore, the catalytic activity of many metalloenzymes requires specific electron transfer proteins.

The complexity of the essential supporting systems that drive metallocluster enzymes is often overlooked by traditional synthetic biology and metabolic engineering strategies. Therefore, the functional expression of metallocluster enzymes must overcome several challenges, including: (i) lack of activity in heterologous hosts; (ii) absent/ineffective maturation pathways; (iii) poorly characterized cofactor requirements or absent/ineffective electron transfer pathways; and (iv) oxygen sensitivity. Traditional strain engineers are often unfamiliar with these complications, which frustrates the development of microbial cell factories that rely on metalloenzyme catalysis. 

#### 2.2.1. The Functional Expression of Metallocluster Enzymes Requires Specific Maturation Pathways

Specific post-translational modifications mediated by specialized proteins [27] are essential for the maturation of metallocluster enzymes. For instance, the functional expression of most FeS cluster enzymes [28], nitrogenases [29], or [NiFe]- and [FeFe]- hydrogenases [25] involve several helper proteins that assemble and deliver the clusters to the apoenzyme via sequential ligand exchange reactions. Heterologous hosts that lack these essential pathways will be unable to functionally express such foreign metallocluster enzymes [30]. Moreover, even when compatible maturation pathways are present in the host, they may not be optimal for a given heterologous enzyme. At least three different FeS cluster maturation systems (called ISC, SUF and NIF) are found in prokaryotes. The function, protein composition and distribution of each system can differ across bacterial species [31]. Within each system, the individual steps required to assemble and deliver FeS clusters are executed by different enzymes. In the *E. coli* ISC pathway, reduced elemental sulfur is generated from cysteine by the cysteine desulfurase IscS and delivered to a cluster scaffold protein, IscU. In the SUF pathway, SufS generates elemental sulfur, while the SufBCD complex serves as the site of FeS cluster assembly [32]. The expression of FeS maturation pathways may also vary and be adapted to growth and environmental conditions. For instance, in *E. coli,* the protein ErpA delivers FeS clusters to the 4Fe-4S enzyme IspG in the presence of oxygen, while IscA mediates this delivery under anaerobic conditions [33].

Furthermore, the maturation of enzymes that contain multiple metalloclusters and/or protein components is far more complex, as multiple maturation steps must be coordinated. For instance, the Mo nitrogenase of *Azotobacter vinelandii* is a two-component enzyme comprised of (i) an Fe protein reductase (NifH) containing a [4Fe4S] cluster; (ii) a catalytic MoFe protein (the α_2_β_2_-tetramer NifDK) containing four complex metalloclusters: two M-clusters (or FeMo cofactor), and two P-clusters (Figure 1b). Therefore, multiple helper proteins are essential for its maturation and catalytic function. The maturation of the reductase NifH is ensured by the maturase NifM, and proteins NifUS for FeS cluster biosynthesis [29,34]. The in-situ assembly and maturation of the P-clusters in NifDK is a stepwise process performed by the proteins NifHZ. P-cluster assembly in NifDK is essential for the subsequent insertion of M-clusters. The biosynthesis and insertion of M-clusters is a highly complex process performed by the coordinated action of at least seven helper proteins [35]. The optimal expression and proper functioning of all these components are required for Mo nitrogenase catalysis in heterologous hosts.

Finally, some catalytic metalloclusters are sensitive to oxidative stress (e.g., IspG and IspH) [36] or O_2_ inactivation (e.g., hydrogenases) [25]. Therefore, such enzymes will require additional proteins for the protection and/or (re)activation of their catalytic metalloclusters.

#### 2.2.2. Metalloenzyme Catalysis Often Requires Specific Electron Transfer Proteins

Many metallocluster enzymes are electronically coupled to small molecule reduced cofactors (i.e., NADH, NADPH, FAD) to sustain catalytic turnover. In some cases, these cofactors can directly deliver electrons to the enzyme, but the large majority of metalloclusters receive electrons via specific electron transfer proteins (ETPs), such as ferredoxins, flavodoxins or reductases [25,37]. The efficiency of electron transfer between protein partners is highly dependent on specific protein–protein contacts, redox potentials and protein concentrations [37]. Because the activity of many metallocluster enzymes is directly coupled to the abundance and efficiency of compatible ETPs, insufficient electron supply constitutes a major limitation to pathway optimization. For instance, the methylerythritol phosphate pathway (MEP) pathway for isoprenoid biosynthesis is known to be limited by the activity of two 4Fe-4S enzymes, IspG and IspH [13]. IspG/H both require an effective reducing partner that shuttles electrons from NADPH via a specific ETP [38]. Diverse studies have demonstrated that electron supply to these enzymes is a limiting factor in increasing the flux through the MEP pathway [38,39]. Insufficient electron transfer to FeS enzymes IspG and IspH could explain why the mevalonate pathway is still favored by biotechnologists for engineered isoprenoid synthesis, despite the higher theoretical carbon yield of the MEP pathway [40].

Moreover, the requirement for specific ETPs can prevent the functional expression of heterologous metallocluster enzymes. For instance, it has been demonstrated that the activity of IspG orthologs in *E. coli* is limited by the need for taxa-specific ETPs [30]. Similarly, different ETPs and cofactors are involved in nitrogenase activity depending on their genomic and metabolic backgrounds [41]. However, the identification of ETPs that support the activity of a heterologous metallocluster enzyme can be a challenging task, as most genes that encode them are not adjacent to metalloenzyme genes [30]. Identifying compatible ETPs is particularly crucial for ensuring enzyme activity in heterologous hosts, as strain engineers need to ensure that compatible ETPs are present in the host of choice. As the functional compatibilities between ETPs and metallocluster enzymes remain poorly characterized, their transferability across different organisms is often unpredictable. Therefore, difficulties in identifying efficient ETPs to sustain the activity of relevant metalloenzymes remain a major barrier to their full exploitation in microbial biosynthesis.

## 3. Strategies to the Promote Abundance of Functional Metallocluster Enzymes in Production Hosts

**Figure 2:**
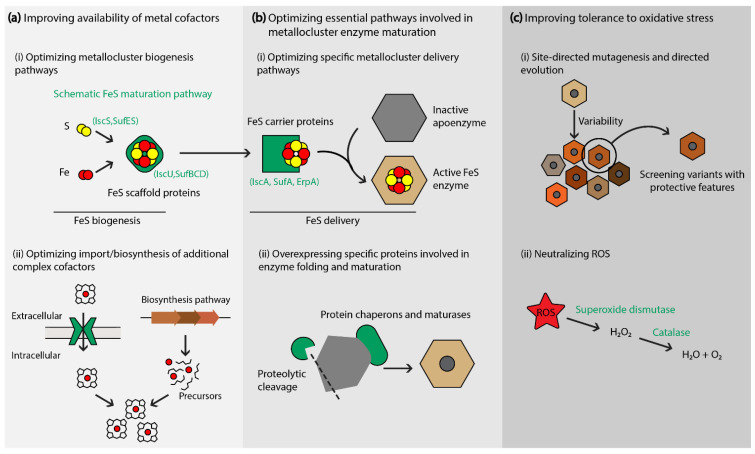
Strategies to enhance metallocluster enzyme maturation and stability. (**a**,**b**) A schematic representation of prokaryotic FeS maturation is presented as an example of the different steps and specific proteins involved in metallocluster enzyme maturation. The availability of metal cofactors can be improved by optimizing their biosynthesis or cellular import (**a**), or by optimizing essential pathways involved in post-translational modifications and protein maturation (**b**). (**c**) Reducing effects of oxidative stress.

Strategies that enhance the peripheral molecular systems involved in metalloenzyme activity—maturation and electron transfer pathways—have been proven effective (and often necessary) in overcoming the challenges related to metabolic pathways that rely on recombinant metallocluster enzymes. The production of active metallocluster enzymes relies on the efficient synthesis and insertion of the metallic cofactor(s) into apoenzyme proteins [27]. The dedicated processes that assemble, deliver, and insert metallic clusters are coordinated by diverse helper proteins. Hence, the production of functional metalloenzymes requires the co-expression of helper proteins that ensure their maturation, activation and catalytic stability (Figure 2).

### 3.1. Optimizing Specific Maturation Pathways

Metalloenzyme maturation pathways are multi-protein systems that execute sequential steps coordinated through specific protein–protein interactions [42]. Such protein systems may be highly specific to the particular metallocluster enzyme and are thus unlikely to be found in heterologous hosts. In such cases, the co-expression of the required maturation pathways in the chassis of choice is the most direct way to ensure the maturation of a given enzyme. For instance, [Fe-Fe]- and [NiFe]-hydrogenases rely on a maturation pathway composed of a dozen proteins [25] that mediate subunit assembly, cofactor incorporation, and in some cases, proteolytic processing [43]. Regardless of this complexity, functional expression of heterologous hydrogenases from diverse organisms is possible when co-expressed with their respective maturation proteins in *E. coli* [44,45,46,47]. Similarly, the functional expression of a heterologous nitrogenase in *E. coli* was first achieved by transferring the full *nif* cluster from *Klebsiella pneumoniae*, which comprises all genes involved in nitrogenase maturation [48]. Recently, another study identified the minimal protein requirements for heterologous metallocluster biosynthesis and maturation of an engineered FeFe nitrogenase system in *E. coli,* comprised of only eight genes [49]. Both heterologous H_2_ biosynthesis and nitrogen fixation illustrate the success of co-expressing maturation pathways in achieving foreign metalloenzyme activity. However, the identification and expression of the required proteins may not be straightforward, hence still limiting the transfer of many interesting metallocluster enzymes [50].

On the contrary, some maturation pathways are ubiquitous and highly conserved across bacterial phyla. This is the case for the maturation pathways of FeS enzymes, which are essential to most cellular organisms (Figure 2). In a given prokaryote, these pathways can be encoded by up to three operons called SUF, ISC, or NIF. While FeS enzymes have an absolute requirement for FeS maturation pathways, compatible systems may be provided by the heterologous host. Although the different FeS cluster assembly and delivery systems rely on specific protein–protein interactions, their components show some promiscuity as they ensure the maturation of different FeS enzymes within the same species (up to 150) [28], and in some cases heterologous FeS enzymes [30,51]. The versatility of FeS maturation pathways notably enables the import FeS orthologs with improved kinetic properties, as demonstrated by the improved isoprenoid production resulting from the functional expression of IspG/H from *T. elongatus* in *E. coli* [39]. Therefore, strategies to boost the host’s FeS maturation pathways have been proven successful in optimizing pathways that rely on FeS enzymes. The enhanced expression of the *isc* operon, either by direct overexpression of the native *isc* genes [52] or by deletion of the negative regulator IscR [39], has been used for instance to improve the activities of IspG/H from the MEP pathway in *E. coli*. The production of antiviral molecules in *E. coli* by diverse prokaryotic Viperins also required the overexpression of the native *isc* operon by deletion of iscR [21].

However, many FeS enzymes lose their activity when heterologously expressed, indicating that the promiscuity of FeS maturation pathways does not necessarily extend beyond all species boundaries. Therefore, the construction of chassis strains that express heterologous FeS maturation pathways can be a strategy to restore the function of foreign FeS enzymes. For instance, the over-expression of the ISC operon from *Azetobacter vinelandii* is a common approach used by biochemistry groups to ensure functional expression of foreign FeS enzymes [16,47,49]. Yet, using the *A. vinelandii* pathway is not sufficient to deliver FeS clusters to all FeS. Little is known about functional versatility of FeS maturation pathways across different organisms, as the protein components and mechanisms required for FeS assembly and incorporation can differ across species [28]. However, a recent study demonstrated that the co-expression of heterologous FeS maturation pathways effectively increases the phylogenetic range of foreign FeS enzymes that can be functionally expressed. Indeed, the expression of the SUF operon from *B. subtilis* not only restored the function of *B. subtilis* NadA in *E. coli*, but also activated eight NadA orthologs from diverse phylogenetic origins [30]. Consequently, the expression of FeS maturation pathways may often broaden host compatibility with certain heterologous FeS enzymes. The cross-species compatibility of FeS maturation pathways also remains to be systematically explored. Furthermore, as all maturation pathway components should be expressed at an appropriate stoichiometry relative to other components, optimizing the abundance of each maturation protein may present an additional challenge for expressing heterologous maturation pathways. Further studies are required to determine the functionality of specific maturation pathways in heterologous hosts, especially pathways that require refactoring due to codon optimization.

### 3.2. Increasing the Synthesis of Additional Essential Metal Cofactors

In addition to metal clusters, additional complex metal cofactors—such as hemes or vitamins—may be required for catalytic activity. Such cofactors are not available in many popular chassis strains or are produced at insufficient levels for high pathway performance. Engineering and optimizing cofactor import/biosynthesis in host strains is therefore an efficient approach to enable/improve the activity of metallocluster enzymes that rely on them. For instance, the heme supply in *E. coli* was improved by optimizing the endogenous production of its precursor 5-aminolevulinic acid (ALA), which subsequently enhanced the activity of heterologous heme-containing enzymes [53]. Another example is the need of cobalamin (vitamin B_12_) for the activity of diverse radical SAM FeS methyltransferases. The activity these enzymes can be enhanced by co-expressing cobalamin import and trafficking pathways [54], such as the *btu* operon from *E. coli*, which enabled the functional expression of *S. laurentii* TsrM in *E. coli* [30]. Alternatively, engineering hosts for de novo biosynthesis of cobalamin could be an approach to optimize pathways that rely on B_12_-dependent FeS enzymes [55].

### 3.3. Improving the Tolerance of Metallic Clusters to Oxidative Stress

The stability of some metallocluster enzymes is greatly hindered by oxidative stress caused by molecular oxygen and/or reactive oxygen species (ROS). The catalytic inactivation of hydrogenases by O_2_, is considered as the major limitation in their biotechnological use for hydrogen production. Similarly, oxidative stress was identified as one of the barriers to the functionality of the bacterial FeS enzymes IspG and IspH in yeast [56], implying that cluster stability after initial FeS cluster assembly is also a limiting factor in metalloenzyme transferability.

Nature has evolved specific amino-acid features and structural arrangements that improve the tolerance of metallic clusters to oxidative damage and that are essential to their stability in aerobic conditions [57]. For a metallocluster enzyme of interest, selecting analogs that have naturally evolved protective features against oxidative stress can be a strategy to stabilize activity in foreign organisms and aerobic conditions. For instance, the O_2_-tolerant membrane-bound [NiFe] hydrogenase (MBH) from *Ralstonia eutropha* holds a special [4Fe-3S] cluster that enables the enzyme to reduce O_2_ into harmless water [58]. Heterologous H_2_ production has been achieved in aerobic conditions by overexpressing MBH homologs in *E. coli*. Cultures expressing functional MBH from *Hydrogenovibrio marinus* increased hydrogen production by two-fold compared to cultures overexpressing the oxygen sensitive hydrogenase Hyd-1 from *E. coli* [59]. Additionally, site-directed mutagenesis and directed evolution can be a reliable method to evolve such protective features. The identification of key residues involved in oxygen sensitivity of the [NiFe] hydrogenase from *Desulfovibrio fructosovorans* enabled the design of a mutated variant with improved O_2_ tolerance and faster reactivation times [60]. In combination with structural and mechanistic studies, these techniques could lead the rational design of enzymes with improved oxygen tolerance without compromising protein function.

Moreover, some metallocluster proteins are protected from oxygen damage by specific helper proteins found in native organisms, which can be co-expressed in heterologous hosts to improve metallocluster stability. For example, the Shethna Protein II [61] and Anf3 oxidase [62] from *A. vinelandii* protect nitrogenase activity from inactivation by oxygen. Additional strategies could rely on the co-expression of enzymes that neutralize ROS, such as superoxide dismutases or catalases, which are known to protect FeS clusters in aerobic organisms [63]. The optimization of natural FeS cluster repair systems in chassis strains could be investigated to improve the performance of FeS enzymes. Expression of metalloenzymes in mitochondria may also protect oxygen-sensitive enzymes from damage, as discussed further below.

## 4. Optimizing Electron Transfer Pathways to Boost Redox Active Metallocluster Enzymes

**Figure 3:**
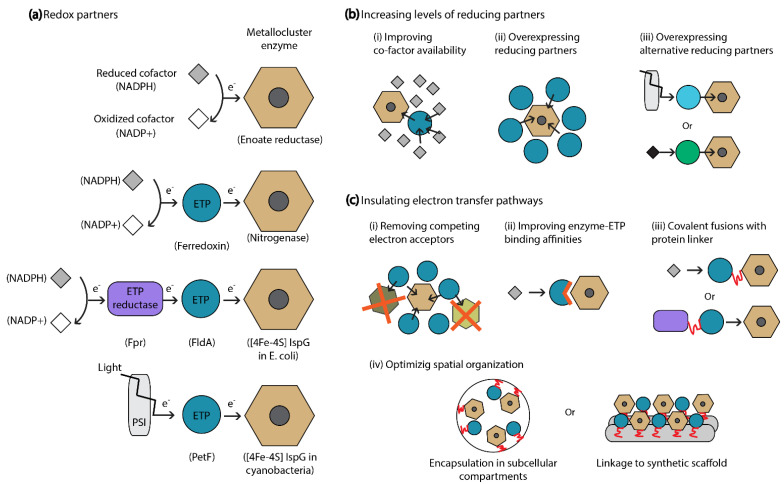
Strategies to enhance electron supply to redox active metallocluster enzymes. (**a**) Schematic representation of the diversity of electron transfer pathways coupled to the activity of different metallocluster enzymes. Most enzymes are coupled to one or multiple ETPs that shuttle electrons from cellular cofactors or photosynthetic chains (such as PSI). Examples of natural redox partners involved in each pathway are given in parenthesis. (**b**) Increasing levels of natural or alternative reducing partners is a successful strategy to enhance electron supply to a given metallocluster enzyme. (**c**) Strategies to insulate electron transfer pathways can be employed to optimize and direct electron flow.

Because the activity of redox active metallocluster enzymes relies on efficient electron supply, optimizing electron transfer pathways linked to biosynthetic enzymes can be an effective strategy to improve pathway performance (Figure 3). This approach is especially relevant when cluster-containing recombinant enzymes display no/low activity in the production host or when direct manipulations of a metabolic pathway (e.g., balancing multi-enzymatic expression) are proven insufficient to further increase product synthesis. To this purpose, the identification, production, and optimization of essential redox partners is crucial.

### 4.1. Identification of Redox Partners Required for Metallocluster Enzyme Activity

To sustain the activity of a heterologous redox active metalloenzyme, it is necessary to ensure that compatible ETPs and reduced cofactors are present in the host. Diverse in vitro assays have been successfully employed to identify the small-molecule cofactors and ETPs that are naturally coupled to metallocluster enzymes. For certain enzymes found in secondary metabolic pathways, the genes that encode partner ETPs can often be within the same biosynthetic gene cluster. If the specific partner ETPs are unknown or are not contained within the same biosynthetic gene cluster, a bioinformatics analysis could help identify potential redox partners in the genomes of native or heterologous organisms. For instance, putative electron delivery systems needed for nitrogenase activity have been identified through genome mining [41]. However, while prokaryotic genomes usually encode multiple ETPs, no universal bioinformatics method to identify partner ETPs for a given enzyme currently exists. Yet, since ETPs display some functional promiscuity and can mediate electron transfer to a diversity of interacting proteins, cellular assays have been successful in identifying compatible ETPs for target enzymes [30,37]. These studies demonstrate that both binding specificity and reduction potentials determine the favorable interactions between ETPs and heterologous metallocluster enzymes. For instance, the expression of ferredoxins from diverse species can support the activity of heterologous IspG homologs from different organisms in *E. coli* [30]. This functional versatility can be an advantage in optimizing electron supply to relevant biosynthetic metallocluster enzymes.

### 4.2. Increasing the Amount of Redox Partners Enhances Electron Supply and Catalytic Activity

#### 4.2.1. Improving Availability of Small-Molecule Cofactors

A straightforward approach to increase electron supply to an enzyme of interest is to optimize the regeneration and availability of its small-molecule redox partner(s) [64]. For instance, strategies that improve NADPH pools can result in increased performance of pathways limited by NADPH-dependent FeS cluster enzymes [38]. The deletion of an enzyme that consumes NADPH in *E. coli*, coupled with expression of a NADH kinase from *S. cerevisiae*, boosted the metabolic flux towards IspG/H enzymes of the MEP pathway.

#### 4.2.2. Overexpression of Partner ETPs to Direct Electron Flow to Metallocluster Enzymes

More targeted approaches to increase the electron flow through given biosynthetic metalloenzymes involve the manipulation of specific redox chains composed of partner ETPs. Because ETPs are genetically encoded, synthetic biology tools can be employed to control their expression, improve interaction affinities with protein partners, or control their allosteric conformations [37].

Approaches that boost the expression of partner ETPs enhance electron supply to specific metallocluster enzymes and improve pathway performance. For instance, overexpression of FldA and Fpr, the partner ETPs of the FeS enzymes IspG/H from the MEP pathway in *E. coli*, improved titers of an isoprenoid product by three-fold [38]. In cyanobacteria, the activity of IspG/H is modulated by a different set of ETPs: the ferredoxin PetF and its associated ferredoxin-NADP+ reductase PetH. Isoprene production was increased by 1.5-fold in *Synechocystis sp. PCC 6803* resulting from the overexpression of PetF/H [65]. Improved metabolic flux through the MEP pathway in *E. coli* was achieved by the heterologous co-expression of IspG/H from *Thermosynechococcus elongatus* combined with PetF/H from the same organism (2.7-fold improvement) [39].

The ability of ETPs to mediate electron transfer between different partner proteins, and their functionality across a wide range of hosts, make them useful tools for engineering synthetic redox chains that drive heterologous metalloenzyme activity [66,67]. For instance, a recent study explored the cross-species compatibility of diverse IspG orthologs with heterologous electron transfer proteins obtained from different species in *E. coli* [30]. The results show that IspG orthologs exhibit activity with multiple ETPs, and vice-versa. This study also identified ETPs from *Streptomyces cattleya* that sustain the activity of the rSAM FeS enzyme TsrM from *Streptomyces laurentii* in *E. coli*, highlighting the importance of expressing surrogate ETPs. Another study identified housekeeping ETP orthologs of NifJ and NifF to partially support an artificial nitrogenase system in *E. coli*, a surrogate electron transport pathway that could be further optimized [49].

Finally, the overexpression of ETPs can lead to excessive redox activities and to the accumulation of reactive oxygen species, causing global imbalances that hinder cell viability and production [37]. Therefore, it is important to tightly regulate the expression of reducing partners (relative to their target enzymes) to optimize electron flux without compromising cell viability.

### 4.3. Insulation Strategies for Electron Transfer Pathways

#### 4.3.1. Removing Competing Reactions

Increasing electron flow to specific metalloenzymes can achieved by preventing electron transfer to other biochemical processes in the cell. An approach to redirect electron flow into a pathway of interest is to remove competing electron acceptors from the host. Agapakis et al. increased hydrogen production in *E. coli* by 40% by deleting a gene encoding a protein that was suspected to interact with the synthetic electron transfer pathway of [Fe-Fe] hydrogenases [46]. Moreover, as protein–protein binding affinities control electron flow between redox partners, targeted mutations in ETPs can reduce or suppress their interaction with competing binding partners, hence redirecting electron flow to biosynthetic metalloenzymes and improving product yields [43,61]. For instance, targeted mutations of a hydrogenase at the interaction surface to enhance the charge-complementarity with a partner ferredoxin improved hydrogen production up to two-fold, compared to the wild-type hydrogenase [46].

#### 4.3.2. Covalent Fusions with Redox Partner

Other methods to enhance electron transfer between desired protein partners are based on increasing their relative local concentration within the cell. Indeed, incrementing the chance of contact between enzymes and ETPs is an efficient strategy to direct the flux into biosynthetic pathways of interest. Improved enzymatic activity and pathway performance can be achieved by directly fusing metalloenzymes to their reducing partner using genetic engineering tools. Such chimeric protein complexes can be synthetically constructed by fusing the desired partners through protein linkers. For instance, the direct fusion of the hydrogenase from *C. acetobutylicum* with a ferredoxin resulted in a 4.4-fold increase in hydrogen production [46]. However, it is difficult to predict how these covalent fusions will impact enzymatic activity [46].

The physical linkage of metalloenzymes with electron carriers can also be employed to engineer alternative electron transfer pathways with improved efficiency. For instance, alternative electron donors have been engineered in *E. coli* to uncouple the activity of the [NiFe]-hydrogenase-3 (Hyd-3) from its natural electron donor formate. An engineered Hyd-3 covalently attached to a ferredoxin from *Thermotoga maritima* accepts electron from pyruvate instead, and sustains in vivo hydrogen production when co-expressed with a pyruvate-ferredoxin oxidoreductase (PFOR) [68].

#### 4.3.3. Spatial Organization of Enzymes and Partner ETPs

Since the physical interaction between partner proteins is critical for electron transfer, their spatial organization can be optimized to enhance electron flux through desired metabolic pathways. Optimized spatial organization of electron donor and acceptor proteins increases their chance of physical contact, and reduces cross-talk with other cellular pathways [69]. Molecular scaffolds can be designed to attach desired redox partner proteins and increase pathway yields in vivo. This approach has been applied to optimize the spatial organization of hydrogenases and ferredoxins to improve hydrogen production in *E. coli*. Attachment of a hydrogenase and a ferredoxin on a synthetic protein scaffold improved hydrogen production by three-fold [46]. The use of rationally designed RNA structures with protein-docking sites to optimize spatial organization and electron transfer boosted hydrogen yields by 50-fold [70]. Other strategies based on subcellular compartmentalization to encapsulate metabolic pathways can be employed to enhance electron transfer and pathway performance [71].

## 5. Towards Transferring Metallocluster Enzymes from Bacteria to Eukaryotes

Eukaryotic organisms, such as yeast or microalgae, are essential hosts in the development of microbial cell factories. As many industrial bioprocesses use eukaryotic microbial hosts, implementing biotechnologically relevant pathways in these organisms is crucial to the large-scale production of chemicals. Unfortunately, attempts of transferring pathways that rely on the activity of bacterial metallocluster enzymes into eukaryotes have encountered limited success. For instance, many bacterial FeS enzymes involved in industrially relevant pathways display little-to-no activity when expressed in yeast, and common synthetic biology interventions have failed to improve their activity [63]. Attempts to engineer the MEP pathway [72], the Entner–Doudoroff pathway [73], or the Weimberg pathway [74] in yeast have met limited success due to the poor activity of bacterial FeS cluster enzymes. The specific challenges encountered when expressing bacterial metalloenzymes within eukaryotes are more fully explored in reference [63], but are briefly discussed here.

The barriers to the functional expression of recombinant metallocluster enzymes in eukaryotic organisms are analogous to those that exist between bacterial species: (i) inefficient maturation and/or electron supply due to incompatibilities between phyla-specific systems; and (ii) oxidative stress. The co-expression of bacterial FeS maturation and reducing pathways in yeast is unfortunately insufficient to enhance enzymatic activity, as demonstrated for the enzymes IspG and IspH [75]. Other strategies focused on disturbing the host’s iron regulation to increase FeS cluster availability have been successfully employed to improve IlvD activity and isobutanol production [76]. Finally, the compartmentalization of bacterial FeS enzymes in yeast organelles (i.e., mitochondria or chloroplasts) remains another promising strategy to sustain their activity in eukaryotes. Indeed, these organelles contain FeS maturation pathways that resemble bacterial systems, as well as compatible ETPs to support catalysis [77]. Moreover, compartmentalization significantly reduces oxidative stress of metallocluster enzymes, hence increasing their catalytic stability [11]. While these strategies are promising, they remain insufficient to the development of viable yeast bioprocesses and additional research is required.

Engineering nitrogen fixation in eukaryotes by expressing bacterial nitrogenases has been a long-sought goal due to the cost of synthetic nitrogen fertilizers; however, nitrogen fixation in plants for self-fertilization has not been achieved due to difficulties in expressing functional nitrogenase systems [78]. Nevertheless, nitrogenase expression in eukaryotes serves as a case study that demonstrates the progress made through the use of many approaches described in this review. The oxygen sensitivity of the nitrogenase maturation pathway was circumvented by targeting expression to the mitochondria [79]. Highlighting the importance of identifying specific electron transfer proteins, a recent study used an *E. coli* cell chassis to determine that electron transfer pathways from chloroplasts, but not mitochondria, are capable of supporting bacterial nitrogenase systems [80]. More recently, 32 orthologs of an iron reductase (NifH) were expressed in tobacco mitochondria, together with protein cofactors involved with FeS transfer [81]. This work specifically identified Fe-S cluster assembly as a significant barrier to nitrogenase function, underscoring the need for further research.

## 6. Conclusions and Perspectives

The aim of making biology easier to engineer is the central goal of synthetic biology. To this purpose, tools and standardized principles for manipulating DNA have been developed over the past two decades. Genetic engineering technologies have enabled the engineering of a wide range of organisms with novel biological functions, including biotechnologically relevant microbial cell factories. However standard synthetic biology tools focused on genetic manipulation have faced challenges in engineering pathways that rely on many catalytically powerful metalloenzymes. Here, we presented an outline of the obstacles to the functional expression and optimization of heterologous metallocluster enzymes in biosynthetic pathways. We also described novel synthetic biology strategies and investigations that could enable harnessing their full biotechnological potential. Such efforts are focused on overcoming obstacles specific to the functional requirements of catalytic metallocluster enzymes: (i) engineering essential supporting pathways involved in enzyme stability and maturation; and (ii) engineering pathways for electron supply that sustain redox active catalysis. The combination and further development of these strategies could be used to optimize novel microbial cell factories with economically viable yields.

However, major challenges to the full exploitation of these engineering approaches remain, as our fundamental knowledge on the biochemistry and barriers to the heterologous expression of metallocluster enzymes is still very limited. Indeed, synthetic biologists cannot easily predict the transferability of a given metallocluster enzyme, nor anticipate what will be required for their activation in heterologous hosts. More studies of phylogenetic compatibility and functional requirements will help develop intuitive rules for harnessing heterologous metallocluster enzymes. To this purpose, original research at the intersection between biochemistry, metabolomics, functional genomics, and synthetic biology is necessary. For instance, large-scale cellular assays could be developed to screen the in vivo functionality of relevant metallocluster enzymes in foreign hosts, and to address the barriers to their transferability and catalytic activity [30]. Furthermore, such studies could also help developing strategies to transfer bacterial metalloenzymes into industrial eukaryotic hosts.

Moreover, recent advances in synthetic biology have enabled de novo engineering of artificial metallocluster enzymes by incorporating catalytic metallocofactors (including abiotic cofactors) into designed protein scaffolds. These artificial enzymes catalyze a remarkable range of natural and synthetic reactions [82], and their biosynthetic potential is tremendous. Yet, the same challenges encountered with natural metalloenzymes often limit their activity, including electron transfer efficiency [83] and catalytic stability [82]. Strategies to enhance the maturation, stability and biochemistry of natural metallocluster enzymes will likely be of significant relevance for improving artificial or bioinspired catalysts [84]. Moreover, designing custom FeS proteins that mediate tunable electron transfer could be of considerable interest for engineering novel redox chains to support metallocluster enzyme activity [85,86]. For instance, synthetic chemical-dependent ETPs have been recently designed to support electron transfer in vivo, in the presence of the chemical inducer rapamycin [85]. Such protein switches could represent a new class of ETPs for the precise regulation of electron transfer across a wide range of redox potentials with direct applications in metabolic engineering.

Besides their potential for microbial biosynthesis, metallocluster enzymes participate in a wide range of key biological processes across kingdoms, including some of technological importance, such as antiviral [21] or antibiotic resistance [87]. Therefore, the relevance of understanding their biochemistry and addressing the barriers to their activity goes beyond applications to microbial cell factories.

## Figures and Tables

**Table 1:** Biotechnological relevance of metalloenzymes.

Name/Type	Metal Cluster Composition	Compound or Compound Family	Native Pathway	Biotechnological Relevance	Ref.
Nitrogenase	Complex FeS cluster [X–7Fe–C-9S] where “X” is molybdenum, vanadium, or iron	Reduction of N2 to bioavailable NH3	Nitrogen fixation	Agriculture; plant-associated nitrogen-fixing bacteria	[11]
D-xylonate dehydratase	[2Fe-2S] cluster	Xylose catabolism, 1,2,4 butanetriol production	Weimberg pathway	Renewable carbon sources	[12]
IspG, IspH	[4Fe-4S] cluster	Isoprenoid precursors	MEP pathway	Pharmaceuticals, fragances, flavors, biofuels, polymers	[13]
IlvD	[4Fe-4S] cluster	Isobutyraldehyde isobutanol	Isoleucine and valine pathway	Biofuels	[14]
BioB	B12-Radical SAM [2Fe-2S] and [4Fe-4S] clusters	Biotin	Biotin biosynthesis	Food supplements, pharmaceuticals, cosmetics, animal feed	[15]
ThnK, ThnL, ThnP	B12-Radical SAM [4Fe-4S] clusters	Thienamycin and carbapenem derivatives	Thienamycin pathway	Antibiotics	[16]
PoyB, PoyC, PoyD	Radical SAM [4Fe-4S] clusters	Polytheonamide cytotoxins	Polytheonamide biosynthesis	Antibiotics	[17]
Fom3	B12-Radical SAM [4Fe-4S] cluster	Fosfomycin	Fosfomycin biosynthesis	Antibiotics	[18]
GenK, GenD1	B12-Radical SAM [4Fe-4S] cluster	Gentamicin	Gentamicin biosynthesis	Antibiotics	[19]
YtkT (radical SAM/FeS)	Radical SAM [4Fe-4S] cluster	Yatakemycin	Yatakemycin biosynthesis	Antitumor	[20]
Viperin	Radical SAM [4Fe-4S] cluster	Antiviral ribonucleotides	Antiviral defense	Antivirals	[21]
Cob enzymes	Contain [4Fe-4S] clusters	Vitamin B12	Cobalamin biosynthesis	Medical and food industries	[22]
Enoate reductases	[4Fe-4S] cluster	Adipic acid, precursor for Nylon-6,6 polymer	Phenylalanine pathway	Commodity chemicals	[23]
[2Fe-2S] Rieske-type oxygenases	Iron-sulfur Rieske domain and non-heme Fe(II)-binding motif	Hapalindole-type products	Hapalindole biosynthesis	Antimycotic insecticidal	[24]
Hydrogenases	[FeFe]- or [NiFe] active site. Contain multiple Fe-S subclusters	Hydrogen (H2) gas	Hydrogen production	Biofuels	[25,26]

## Data Availability

Not applicable.

## Appendix A

**Table A1:** Crystal structures of metalloenzymes.

Enzyme	Origin	Uniprot ID	PDB ID
Fe-Fe hydrogenase	*Clostridium pasteurianum*	P29166	1FEH
Ni-Fe hydrogenase	*Desulfovibrio fructosovorans*	P18187	1FRF
[2Fe-2S] ferredoxin FdxB	*Pseudomonas putida*	Q76CS9	3AH7
D-xylonate dehydratase	*Caulobacter vibrioides CB15*	Q9A9Z2	5OYN
[2Fe-2S] and [4Fe-4S] rSAM enzyme BioB	*Escherichia coli*	P12996	1R30
Nitrogenase molybdenum-iron protein	*Azotobacter vinelandii*	P07328	6UG0
[4Fe-4S] Viperin	*Nematostella vectensis*	A7RNF3	7N7H
[4Fe-4S] enzyme IspG	*Aquifex aeolicus*	O67496	3NOY
